# Volvulus of sigmoid colon in a challenged adolescent: An unusual case report

**DOI:** 10.1016/j.amsu.2019.06.009

**Published:** 2019-06-18

**Authors:** Tika Ram Bhandari, Sudha Shahi

**Affiliations:** a Department of General Surgery, People's Dental College and Hospital, Kathmandu, Nepal; b Formerly Department of General Surgery, Universal College of Medical Sciences, Bhairahawa, Nepal; c Department of ENT Head and Neck Surgery, National Academy of Medical Sciences, Kathmandu, Nepal

**Keywords:** Sigmoid volvulus, Adolescent, Mentally disabled, Bowel obstruction, Case report

## Abstract

Sigmoid volvulus is very uncommon cause of intestinal obstruction in pediatrics population withhigh rate of mortality. To date, few cases of sigmoid volvulus in children and association with several condition has been reported in literature, of them very few cases are with mental disability. We report a challenged (mentally disabled) 14-year old adolescent boy presented asan emergency with feature of complete bowel obstruction. Abdominal X-rays shows dilated loop of large bowel with inverted U shaped. Volvulus of sigmoid colon was found during laparotomy and successfully managed with resection of a redundant colon with colocolic end to end anastomosis. Sigmoid volvulus is relatively uncommon in children as compared to adults. Surgeons should be attentive of this rare entity, cause of large bowel obstruction to allow for early diagnosis and to enable better patient outcomes by reducing the morbidity and mortality.

## Highlights

• Sigmoid volvulus is very uncommon cause of intestinal obstruction in pediatrics population.
• We report an unusual case of sigmoid volvulus in mentally disabeled adolescent male.
• Awareness of this a rare cause of bowel obstruction is important for appropriate management in a mentally disabled adolescent.

## 1 Introduction

Volvulus is a rotation of part of the intestine around its mesentery, causing an intestinal obstruction. Sigmoid volvulus is very rare in the children in comparison to adults [1]. The clinical symptoms are often abdominal pain, abdominal distention, and vomiting. Diagnosis may be missed or delayed due to the rarity of this disease in children. Morbidity and mortality related to this is very high due to closed-loop obstruction, bowel ischemia and hypovolemic shock [2]. Early diagnosis and prompt surgical treatment may be a life-saving measure. Association of volvulus with certain conditions like pregnancy, colitis, Hirschsprung's disease, prune belly syndrome, chronic constipation, laxative abuse and congenital redundant colon with long mesentery also have been reported [3]. There are very few reported cases of volvulus in children with mental retardation to date [4,5]. Sigmoid volvulus in mentally disabled patient may be associated with aerophagia and constipation that induce bowel distention. Having a adolescent with mental disability, diagnosis of acute abdomen is often challenging because they tend to present with nonspecific clinical presentation and history cannot be extracted correctly. We report a case of sigmoid volvulus in a mentally disabled adolescent boy and treated with resection and anastomosis. This case has been managed in Universal College of Medical Sciences, Bhairahawa, Nepal Teaching Hospital by consultant General Surgeon and team. This case has been reported according to SCARE criteria for case reports [6].

## 2 Case presentation

**Fig. 1:**
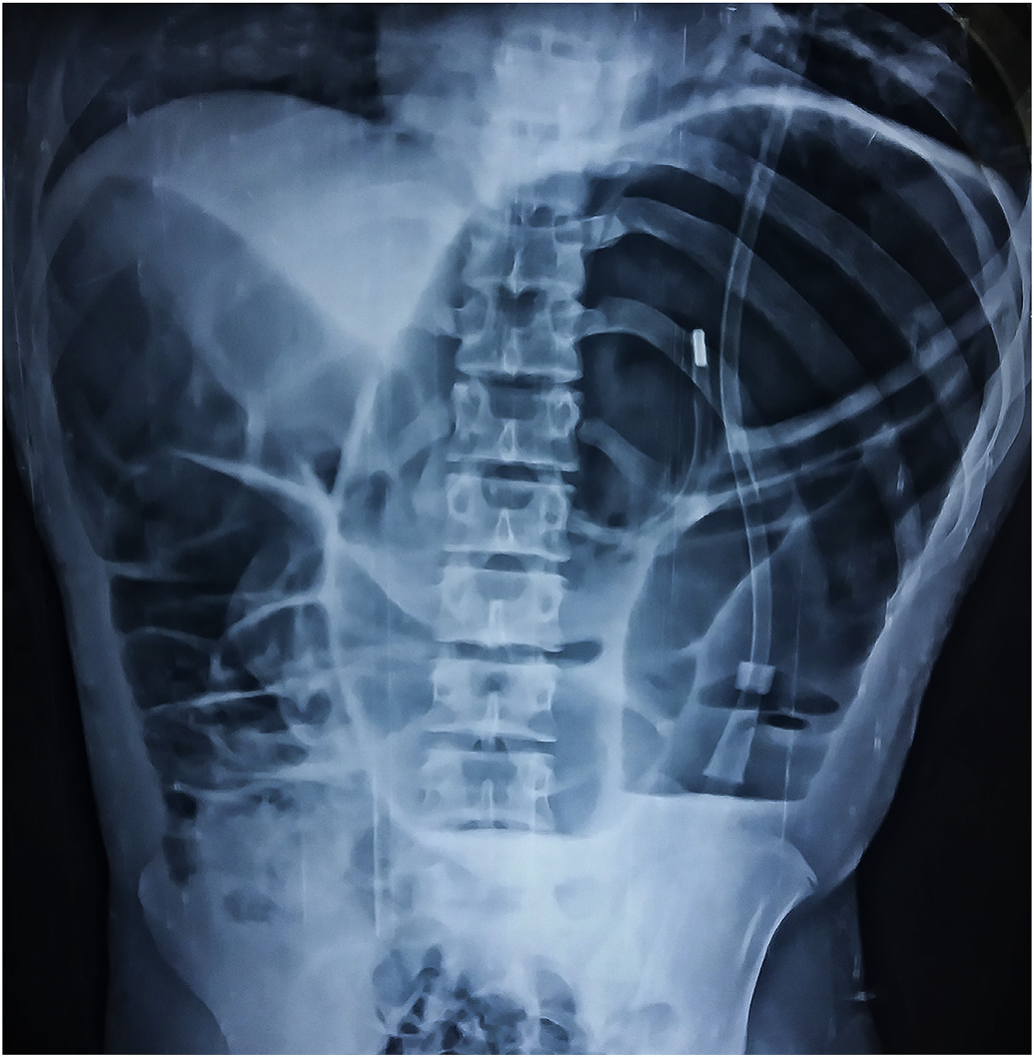
Showing dilated loops of large bowel loop in the left upper quadrant (bent inner tube/omega sign).

**Fig. 2:**
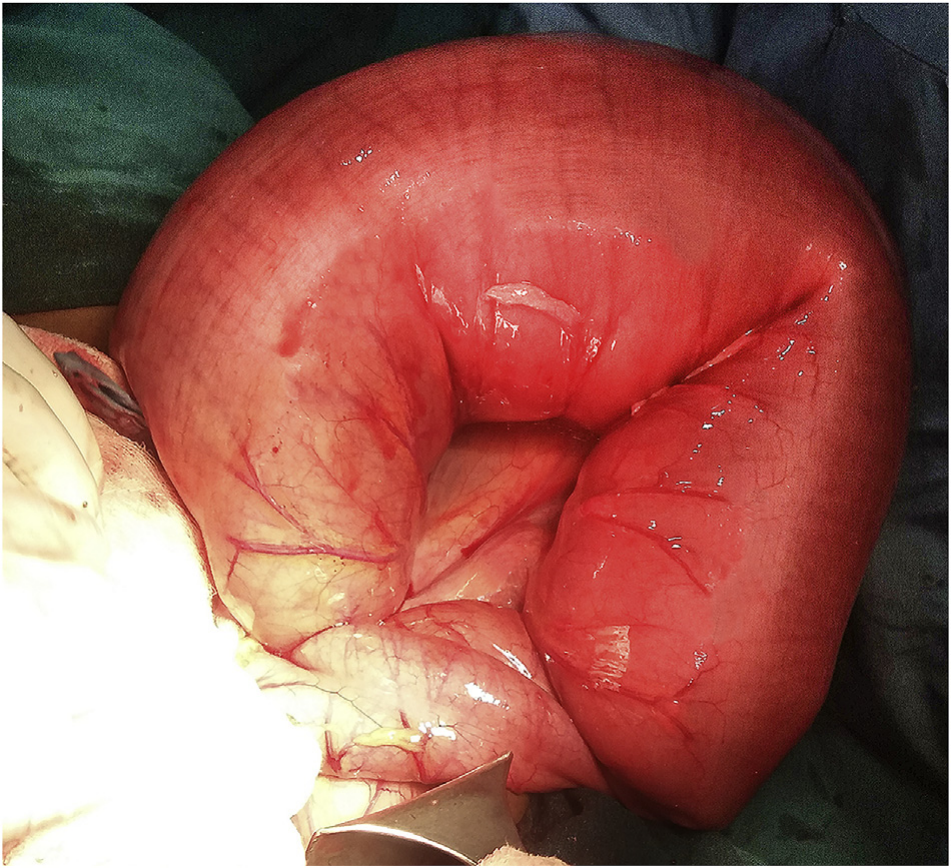
Showing volvulus of the sigmoid colon with clockwise rotation intaoperatively

A 14-year-old boy presented as an emergency with 3-days history of pain in abdomen and distention.The nature of pain was the sharp, severe and non-radiating type. He also complained of nausea and vomiting. There was a history of constipation for which he dad to take laxatives regularly. He has a significant medical history of premature birth at 32 weeks, developmental 3 delay and mental retardation. He denied other past surgery and relevant family history. On examination, the patient was ill looking, dehydrated. He had cold extremities and sunken eyes. Blood pressure was 100/70 mmHg, Pulse rate 110 per minute with low volume. No abnormality was detected in respiratory, cardiovascular and nervous systems. Abdomen examination revealed gross distention, hyper-resonance with mild tenderness. Bowel sounds were sluggish and per rectal examination was unremarkable. Patient resuscitated with a bolus of intravenous fluid (normal saline) antibiotics, analgesics and nasogastric decompression. On investigation, white blood cells were 12,000 cells/mm3, hemoglobin 11 gm/dL platelet count 150,000 cells/mm3, serum sodium 140 mEq/L and potassium 4.2 mEq/L. Plain X-ray abdomen showed dilated loops of large bowel loop in the left upper quadrant (bent inner tube/omega sign) (Fig. 1). The patient was taken for laparotomy with a diagnosis of sigmoid volvulus. During laparotomy, midline incision released 300 mL of serous fluid. A volvulus of the sigmoid colon with 360-degree clockwise rotation was found (Fig. 2). The redundant sigmoid colon was hugely dilated which was resected after detortion and resection anastomosis was performed. The patient was discharged from hospital on 8 th postoperative day. Post-operative period was uneventful.

## 3 Discussion

This case represents an unusual association of sigmoid volvulus in mentally disabled adolescent. Volvulus of sigmoid colon comprises four percentage of all intestinal volvulus in pediatric populations. While approximately one third of elderly patients have history of sigmoid volvulus problem and 50–80% have association with comorbid illness [7]. Salas et al. and their team reported series of 63 cases of sigmoid volvulus in children from 1941 to 2000 [1]. In 1990, Smith et al. [8] also published article on 48 children with sigmoid volvulus. Adding to this, in 1994, Mellor and Drake reported 10 cases of sigmoid volvulus in children out of 14 cases of colonic volvulus from 1955 to 1992 [9]. These studies give a gross idea of how rare the condition is in case of children. Although our patient was adoloscent, the average age of child reported is eight years with male preponderance (male/female ratio 3.5:1) [1]. Patel RV et al. recently reported adolescent girl with sigmoid volvulus presented with complete large bowel obstruction [10]. The incidence of volvulus in adults is higher in Africa, the Middle East, Asia, and Russia which reported as “Volvulus Belt”. However, for pediatric populations higher incidence has been reported from West, including North America [11]. Sigmoid volvulus frequently occurs as acute abdomen as in our case but less commonly its presentation may as a chronic recurrent abdominal pain [12]. The most common symptoms are abdominal pain (66%) and vomiting (31%), while commonest signs are abdominal distention (69%) and tenderness (41%) [2]. The diagnostic delay may lead to bowel ischemia, perforation, shock and multi-organ dysfunction leading to death. Sigmoid volvulus in children has been reported comprising various causative factors [3,13]. Literature on sigmoid volvulus in mentally disabled adolescent with few case have been published. Colinet S et al. recently reported sigmoid volvulus in children associated with mental retardation, myopathy and costipation in their large series [14]. Another study from UK also reported sigmoid volvulus in adolescent girl associated with severe learning disability, constipation, acquired microcephaly, hypotonia and iron deficiency anemia [10]. In our patient, there were several risk factors including chronic constipation, high fiber consumption, habit of eating one large meal and mental retardation. Sigmoid volvulus in mentally disabled patient may be associated with aerophagia and constipation that induce bowel distention. Chronic constipation causes lengthening of the colon, produce a redundant sigmoid colon which is a prerequisite to volvulus. Consumption of high fiber diet may be another factor which causes development of a long redundant colon. The habit of eating one large meal each day also can be considered for the development of sigmoid volvulus in our part of world.

Having a adolescent with mental disability, diagnosis of acute abdomen is often challenging because they tend to present with nonspecific clinical presentation and history cannot be extracted correctly. Radiological imaging studies have an important role in the pre-operative diagnosis of such patients with communication difficulties. Plain erect abdominal X-rays generally show a dilated sigmoid colon and a coffee bean-like shape formed by grossly dilated and closely apposed sigmoid loops [15]. In nongangrenous or nonperforated sigmoid volvulus patient water soluble contrast enema studies can be used for diagnostic and therapeutic purpose. A whirl sign of the dilated sigmoid loop around mesocolon and a bird-beak appearance may characteristically seen in CT abdomen [16]. The radiological findings seen in X-rays was diagnostic in our patient.

The main aim of treatment is to release the bowel obstruction, prevent ischemia and gangrene and prevent recurrence [17]. Although Endoscopic decompression with rectal biopsy followed by definitive surgical therapy after 2–3 days has been mentioned in the literature [17], the accessibility in resource poor hospitals and its relevance in a pediatric population still challenging in developing countries. Thus, reduction of a sigmoid volvulus by barium enema, proctoscopy and rectal tube decompression are common nonoperative measures that we generally performed in cases in which there is patient is hemodynamically stable and no feature of perforation. Recurrence rate of 35–90% with non-operative therapy has been reported [14,18] in the literature and thus, surgical management has been considered by most surgeons. We couldnot performed nonoperative maneuver as our patient was hemodynamically unstable and with positive abdominal tenderness. During surgical treatment, individual risk factors and operative findings are the main factors for determining the choice of operation. There are various surgical options which include derotation alone, derotation with colopexy, resection with primary anastomosis and resection with end-ostomy. We performed exploratory laparotomy and resection of a redundant colon with colocolic end to end anastomosis. Resection of sigmoid and dilated colon with primary anastomosis is choice of operation in children due to its low recurrence rate [19,20]. A minimal invasive elective laparoscopic assisted sigmoidectomy has been reported by Liu et al. without recurrence of volvulus during four year of follow up [21]. Limited number of skilled manpower and training chances, unavailibility of advanced instruments for noninvasive or minimal invasive procedures, and inadequacy of resources to maintain equipment are usual challenging tasks in resource poor settings. Besides that, people usually have poor access to hospital because of poor health knowledge, poor transportation services, economic contraints and long distances in rural areas which ultimately causes of delayed presentation to the hospitals. The present case report also highlights the necessity for larger studies with volvulus of colon and mental disability in adolescent to find the actual relationship of cause and effect.

## 4 Conclusion

The sigmoid volvulus is an unusual entity in mentally disabled adolescent patient. Surgeons should be attentive of this rare entity, cause of large bowel obstruction to allow for early diagnosis and to enable better patient outcomes by reducing the morbidity and mortality.

## Consent for publication

Written informed consent was obtained from the parents for publication of this case report and any accompanying images. A copy of the written consent is available for review by the Editor-in-Chief of this journal.

## Competing interests

All authors declare that they have no competing interests.

## Provenance and peer review

Not commissioned, externally peer reviewed.

## Ethical approval

Written informed consent was signed by the parents.

## Sources of funding

No sources of funding

## Author contribution

1-Tika Ram Bhandari - study concept or design, data collection, literature search, writing paper, final decision to publish.

2-Sudha Shahi – Study concept or design, litreture search, writing paper, final decision to publish.

## Conflicts of interest

No conflict of interest.

## Registration of research studies

Is a case report.

## Guarantor

Dr. Tika Ram Bhandari.
